# Genetically modified "obligate" anaerobic *Salmonella typhimurium* as a therapeutic strategy for neuroblastoma

**DOI:** 10.1186/s13045-015-0196-3

**Published:** 2015-08-19

**Authors:** Zhu-Ling Guo, Bin Yu, Bo-Tao Ning, Shing Chan, Qiu-Bin Lin, James Chun-Bong Li, Jian-Dong Huang, Godfrey Chi-Fung Chan

**Affiliations:** Department of Paediatrics & Adolescent Medicine, Li Ka Shing Faculty of Medicine, the University of Hong Kong, Hong Kong, SAR People’s Republic of China; Department of Biochemistry, Li Ka Shing Faculty of Medicine, the University of Hong Kong, Hong Kong, SAR People’s Republic of China; HKU-SIRI, the University of Hong Kong, Hong Kong, SAR People’s Republic of China; Key Laboratory of Optoelectronic Devices and Systems of Ministry of Education and Guangdong Province, College of Optoelectronic Engineering, Shenzhen University, Shenzhen, People’s Republic of China; Department of Hematology & Oncology of Children’s Hospital, Zhejiang Key Laboratory for Diagnosis and Treatment of Neonatal Diseases, Zhejiang University School of Medicine, Hangzhou, People’s Republic of China

**Keywords:** Anaerobic *salmonella*, Neuroblastoma, Orthotopic mouse model, Immune-compromised host, Macrophage, Cancer treatment

## Abstract

**Background:**

Neuroblastoma currently has poor prognosis, therefore we proposed a new strategy by targeting neuroblastoma with genetically engineered anaerobic *Salmonella* (Sal-YB1).

**Methods:**

Nude and nonobese diabetic-severe combined immunodeficiency (NOD-SCID) orthotopic mouse models were used, and Sal-YB1 was administered via tail vein. The therapeutic effectiveness, bio-safety, and mechanisms were studied.

**Results:**

No mice died of therapy-related complications. Tumor size reduction was 70 and 30 % in nude and NOD-SCID mice, respectively. No *Salmonella* was detected in the urine; 75 % mice had positive stool culture if diaminopimelic acid was added, but all turned negative subsequently. Tumor tissues had more Sal-YB1 infiltration, necrosis, and shrinkage in Sal-YB1-treated mice. Significantly higher expression of TLR4, TNF-stimulated gene 6 protein (TSG6), and cleaved caspase 1, 3, 8, and 9 was found in the tumor masses of the Sal-YB1-treated group with a decrease of interleukin 1 receptor-associated kinase (IRAK) and nuclear factor of kappa light polypeptide gene enhancer in B-cells inhibitor alpha (IκBα). There was a high release of TNFα both in human macrophages and mouse tumor tissues with Sal-YB1 treatment. The antitumor effect of the supernatant derived from macrophages treated with Sal-YB1 could be reversed with TNFα and pan-caspase inhibitors.

**Conclusions:**

This new approach in targeting neuroblastoma by bio-engineered *Salmonella* with the assistance of macrophages indirectly may have a clinical therapeutic impact in the future.

## Background

Neuroblastoma is an aggressive pediatric malignant tumor with high variability in biological and clinical behaviors [1–3]. Despite intensive multi-modality therapies, the outcome of advanced stage neuroblastoma remains extremely poor. Novel therapeutic approach to improve the survival of this group of children is urgently needed.

It is known that cancer stem cells remain viable in the hypoxic tumor core with minimal blood supply. Hence, this affects the drug delivery and has been considered a resistant mechanism to conventional chemotherapy for cancer [4–7]. Attenuated pathogens such as *Salmonella typhimurium* and measles virus have been adopted as a form of biotherapy among the burgeoning anti-cancer strategies [8–10]. However, many of such “bio-bullets” still face the challenge of balance between potential side-effects and anti-cancer effectiveness [11, 12]. Systematically evaluating their effectiveness and safety prior to their actual clinical application to cancer patients is mandatory. To overcome such adversity and also mimic the actual disease characteristics, we proposed to use a genetically engineered anaerobe *S. typhimurium* strain YB1 (Sal-YB1) as a form of biotherapy for the intra-adrenal orthotopic xenograft human neuroblastoma mouse models using either nude or nonobese diabetic-severe combined immunodeficiency (NOD-SCID) mice [13].

The genetically modified Sal-YB1 can only proliferate in hypoxic environment (oxygen <0.5 %) [13]. Otherwise, it must be supplemented with diaminopimelic acid (DAP) when it is cultured under aerobic condition [12, 13]. For bacteria, DAP is an indispensible component in the synthesis of cell wall. In the wild type *Salmonella* strain, DAP is normally developed by the essential gene *asd*. The expression of *asd* is modified and regulated by the hypoxia-conditioned promoter in the Sal-YB1 strain. Compared with the two more commonly used VNP20009 and SL7207 *Salmonella* strain, this genetically modified Sal-YB1 exhibited excellent in vivo cytotoxic effect and had low therapy-related death rate on a human breast cancer model [13].

The orthotopic mouse model has been developed by implanting neuroblastoma into the fat pad of the mice adrenal glands, which is the most common primary site of neuroblastoma in patients. This xenograft model could simulate the actual microenvironment of the tumor. Furthermore, nude mouse together with NOD-SCID mouse model are relatively robust tools to mimic the deficient immune states of patients undergoing chemotherapy [14, 15]. There are specific immune deficiencies found in both the nude and NOD-SCID mouse. For example, the nude mouse cannot generate mature T lymphocytes whereas the NOD-SCID mouse has deficient T- and B-lymphocytes; abnormal natural killer (NK) cells and macrophages (MΦ) both in terms of numbers and function. The neuroblastoma cell line SK-N-LP/luciferase that we used is transduced with the luciferase gene. The bioluminescence is closely correlated to the tumor growth under in vivo imaging system Xenogen 100 (IVIS 100).

In the present study, we chose the new generation Sal-YB1 as the mono-biotherapy for the orthotopic murine models using mice with different immunological backgrounds. Our purpose was to investigate the therapeutic effectiveness and biosafety on both kinds of mice when they were treated with virulent bacteria. The possible immunological mechanism was also explored to guide our clinical application of this potential “bio-bullet”—Sal-YB1.

## Results

### Orthotopic neuroblastoma mouse models with or without Sal-YB1 treatment

**Fig. 1:**
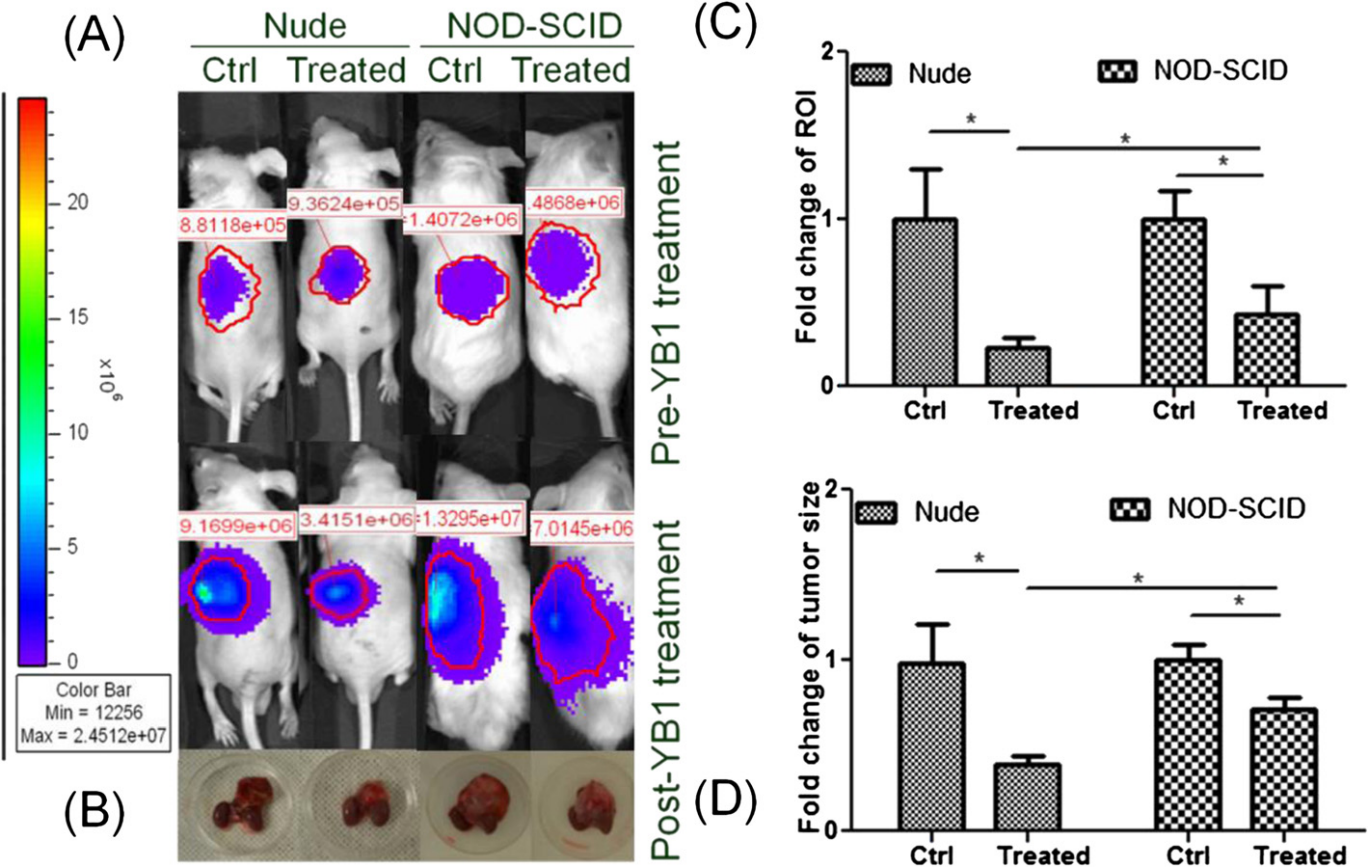
Sal-YB1 could suppress neuroblastoma growth in both nude and NOD-SCID mice. Tumor growth with/without Sal-YB1 treatment was assessed by **a** in vivo imaging system and **b** tumor size measurement. The tumors and kidneys were placed on a 30-mm-diameter dish. **c** Rate of ROI change was significantly different within either nude or NOD-SCID mice and also between these two groups (*P* < 0.05). **d** The *post mortem* tumor size increase rate post-YB1 treatment was reduced to 30 % in nude mice and 70 % in NOD-SCID mice when compared to control (*P* < 0.05). The *error bars* showed means ± SEM for four mice in each group. *P < 0.05 by an unpaired two-tailed *t* test

Ten nude mice and ten NOD-SCID mice were implanted with human neuroblastoma initially. However, before the Sal-YB1 treatment was started, both groups had one mouse each which died due to paralysis. The paralysis was subsequently due to metastasis of orthotopic adrenal neuroblastoma cells to the brain within 2 to 3 weeks. Three-week post-tumor cells transplantation, the nude (*n* = 8) and NOD-SCID (*n* = 8) mice were divided into two groups. At week 2 after inoculation with or without Sal-YB1, all mice survived. Rate of region of interest (ROI) change was significantly different within either nude or NOD-SCID mice and also between these two groups (*P* < 0.05). The *post mortem* tumor size increase rate post-YB1 treatment was reduced to 30 % in nude mice and 70 % in NOD-SCID mice (Fig. 1). Such reduction was mainly due to tumor necrosis as suggested in the following paragraphs.

Mouse body weight and temperature were recorded daily. The urine and stool were also collected daily for 2 weeks after Sal-YB1 was administered. Although the body weight declined in the initial 2 days post-YB1 injection, the nude and NOD-SCID mice of the treated group regained body weight from day 3 onwards. Body surface temperature showed no obvious change during the treatment course. On the first day post-YB1 injection, we could still observe that the mice turned inactive. Rash could be transiently found on the skin of nude mice, but it was relatively difficult to observe in NOD-SCID due to the presence of fur. These febrile reactions might reflect transient bacteremia-triggering immune reaction. However, they have no evidence in inducing organ damage as reflected by the tissues examination when the mice were sacrificed.

### Ex vivo Sal-YB1 side effect evaluation on mouse models

**Fig. 2:**
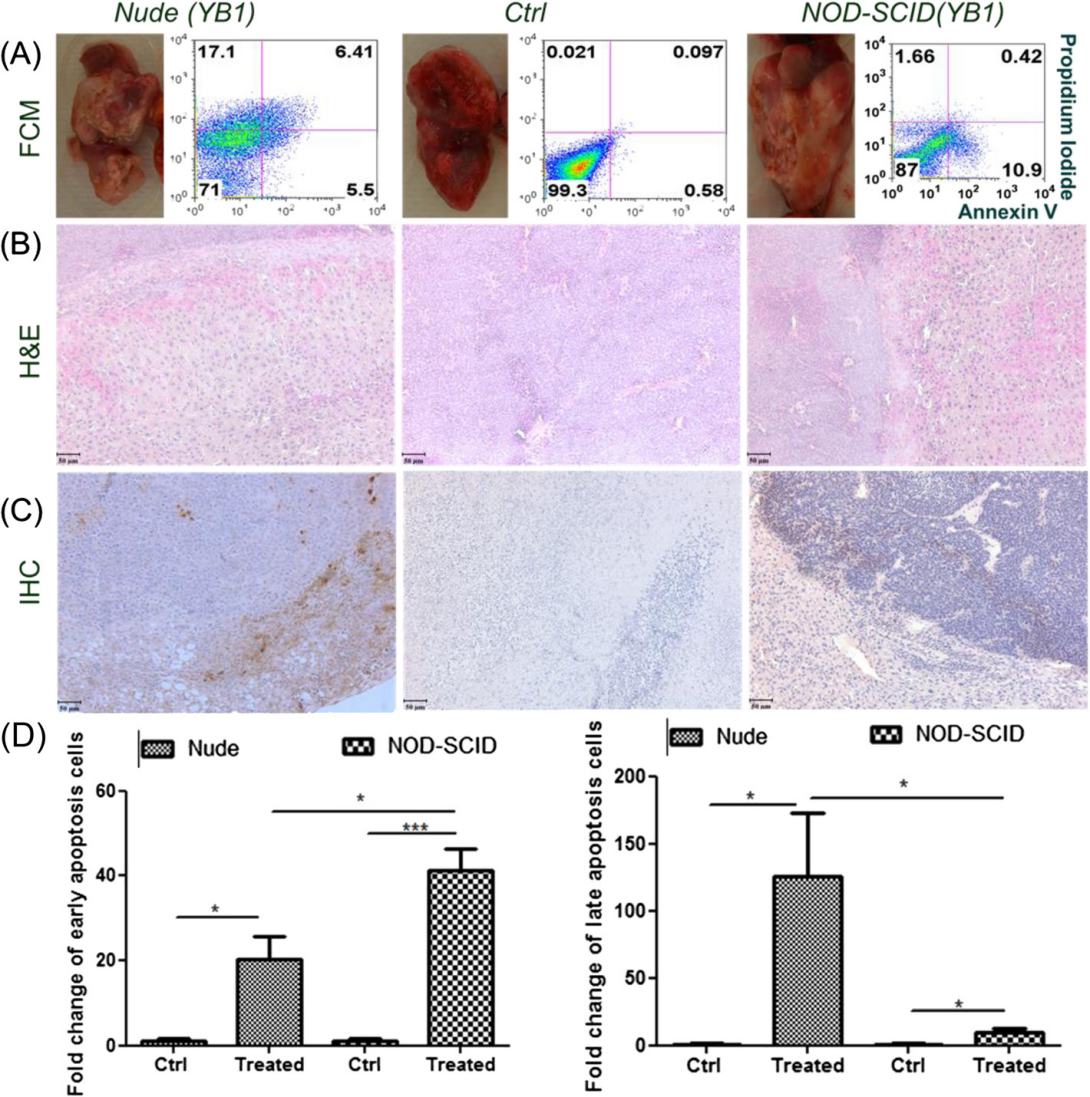
Sal-YB1 could induce cell death in neuroblastoma xenografts of both the nude and NOD-SCID mice. Tumors with YB1-treated and -untreated groups were evaluated by **a** flow cytometry (FCM), **b** hematoxylin and eosin (H&E), and **c** immunohistochemistry (IHC) staining, respectively. *Salmonella* could be detected within the tumor mass. *Scale bar*: 50 μm. **d** Bar diagrams of Annexin V/PI result with statistical analysis. The *error bars* showed means ± SEM for four mice in each group. *P < 0.05 and ^***^ *P* < 0.001 by an unpaired two-tailed *t* test

Flow cytometry, hematoxylin and eosin (H&E), and immunohistochemistry (IHC) staining were applied to evaluate the cytotoxic activities and tissue distribution of Sal-YB1. Sal-YB1-treated tumor tissues showed necrosis, which was tested by annexin V/PI assay and H&E staining (Fig. 2a, b). IHC staining revealed that Sal-YB1 formed clusters within the tumor mass (Fig. 2c) for both nude and NOD-SCID mice without too much differences in their extent of necrosis. It is interesting to note that there are more late apoptotic cells (annexin V+/PI+) for the nude mice but relatively more early apoptotic cells (annexin V+/PI−) for the NOD-SCID mice as found in the annexin V/PI assay. However, the total numbers of apoptotic cells (annexin V+/PI− plus V+/PI+) were more for the nude mice group (Fig. 2d). The findings suggested that tumor in nude mice is more susceptible to Sal-YB1 treatment, reflecting some other factors relating that the immune system might play a role in the anti-tumor effect.

*Salmonella* was not detected in the urine. However, 75 % of the mice had positive Sal-YB1 in their stool. They all turned negative within 2 weeks. Particularly, *Salmonella* was undetectable in stool colony-forming unit (CFU) test without DAP supplement suggesting that these residual Sal-YB1 found in the stool will not be able to survive and proliferate when they were passed under normoxic environment. No matter if mice with tumor implantation or not, both nude and NOD-SCID group did not have Sal-YB1 detected in the heart, spleen, liver, lung, kidney, brain, spinal cord, and blood at 2 weeks post-treatment. However, hepatosplenomegaly was observed in Sal-YB1-treated mice but there was no evidence of either inflammation or necrosis as shown by annexin V/PI and H&E stain.

### Sal-YB1 therapy mechanism on neuroblastoma

**Fig. 3:**
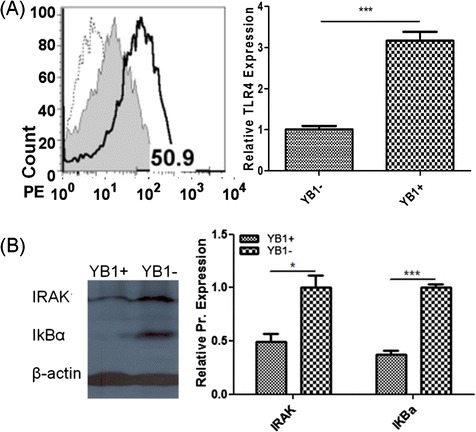
Sal-YB1 could upregulate TLR4 expression and downregulate IRAK and IκBα in tumor tissues. **a** Flow cytometry of TLR4. *Dashed line*, *gray filled* histogram, *continuous line* represented isotype control, YB1-untreated and -treated mice, respectively. Based on the isotype control, TLR4 expression in the YB1-treated mice was gated as 50.9 %. **b** IRAK and IκBα Western blotting. There was a significant difference for IRAK (*P* < 0.05) and IκBα (*P* < 0.001) between the YB1-treated and -untreated groups. The band density of IRAK and IκBα were normalized to β-actin. The *error bars* showed means ± SEM for three nude mice in each group. *P < 0.05 and ^***^ *P* < 0.001 by an unpaired two-tailed *t* test

**Fig. 4:**
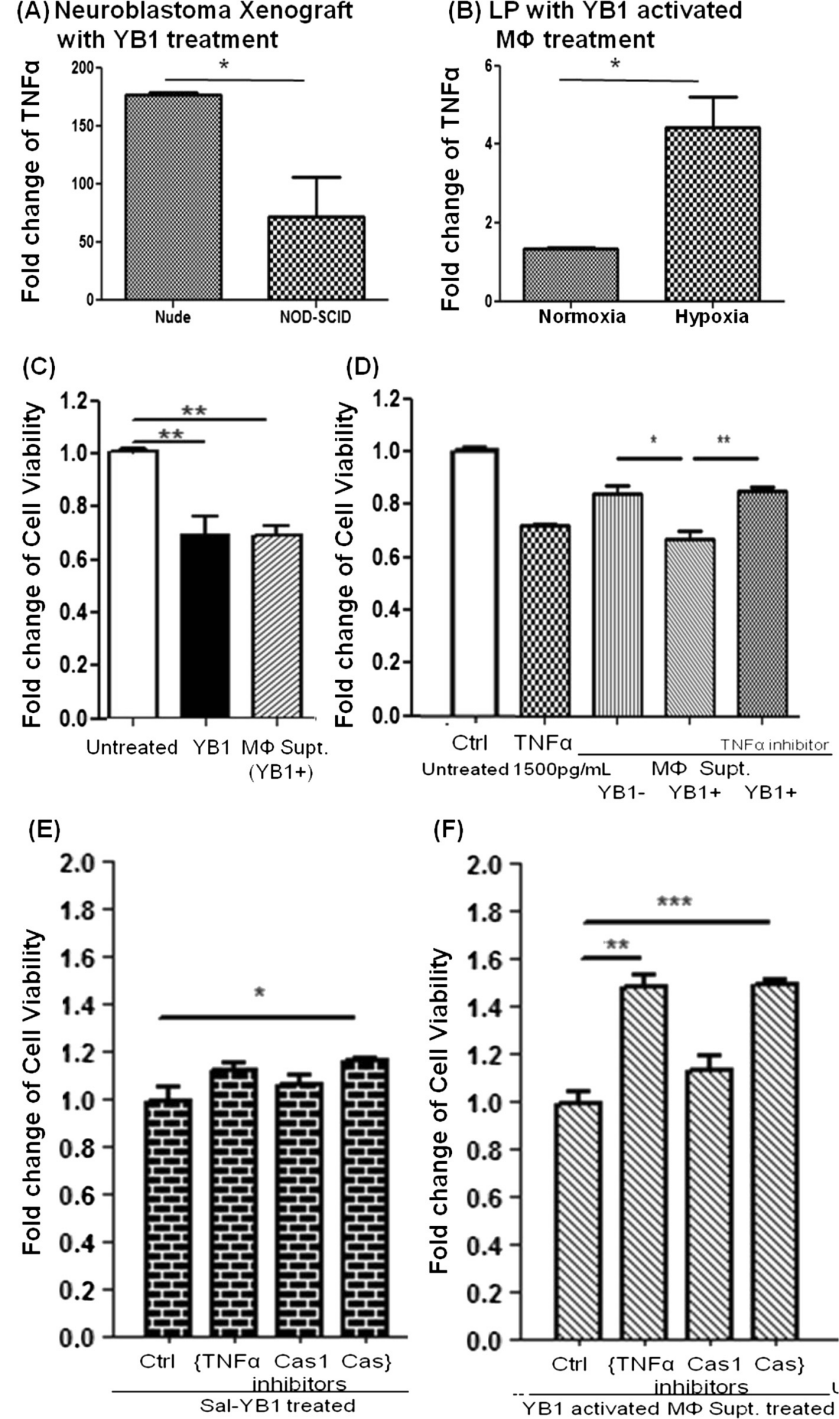
Sal-YB1 could enhance the anti-neuroblastoma effect of macrophages via TNFα. TNFα ELISA assay quantified the Sal-YB1 targeting effect on both **a** mouse tumor tissues and **b** human macrophages. Relatively, the expression of TNFα in nude mice and 2-day hypoxia-cultured macrophages were significantly higher than in NOD-SCID mice and nomoxia-cultured macrophages, respectively (*P* < 0.05). The *error bars* showed means ± SEM for three mice in **(a)** and three healthy human donators in **(b)**. *P < 0.05 by an unpaired two-tailed *t* test. **c** TNFα and pan-caspase inhibitors could reverse the anti-neuroblastoma effect by macrophages. The viability of neuroblastoma was evaluated by XTT test. Pan-caspase inhibitor blocked the apoptosis of neuroblastoma both in Sal-YB1 (*P* < 0.05) and 50 % supernatant of the Sal-YB1 activated macrophages (*P* < 0.001)-treated group. Anti-TNFα significantly rescued neuroblastoma co-cultured with the supernatant from YB1 pre-treated macrophages (*P* < 0.01). The *error bars* showed means ± SEM in triplicate experiments. *P < 0.05, **P < 0.01, and ***P < 0.001 by an unpaired two-tailed *t* test

Sal-YB1 could significantly upregulate Toll-like receptor 4 (TLR4) expression in tumor tissues by flow cytometry. Interleukin 1 receptor-associated kinase (IRAK) (*P* < 0.05) and nuclear factor of kappa light polypeptide gene enhancer in B-cells inhibitor alpha (IκBα) (*P* < 0.001) were downregulated in the xenograft triggered by Sal-YB1 as shown by Western blotting (Fig. 3). There were high release of TNFα from both human macrophage and mouse tumor tissues in the Sal-YB1-treated group as shown by ELISA (*P < 0.05*). The expression of TNFα in nude mice and 2-day hypoxia-cultured macrophages were significantly higher than that of the NOD-SCID mice (Fig. 4a) and normoxia-cultured macrophages (Fig. 4b), respectively. Besides, the Sal-YB1 could only be detected in hypoxia-cultured macrophages by CFU counting. The viability of neuroblastoma was decreased when co-cultured with 50 % supernatant of the YB1 pre-treated macrophages (*P* < 0.01); however, it could be rescued by TNFα inhibitor (*P* < 0.01) (Fig. 4c, d). These data suggested that the endotoxin (LPS) from *salmonella* could activate the TLR4 and the downstream signalling pathway leading to the release of TNFα by macrophages and the TNFα will subsequently induce indirect tumor cell killing in the cancer microenvironment.

**Fig. 5:**
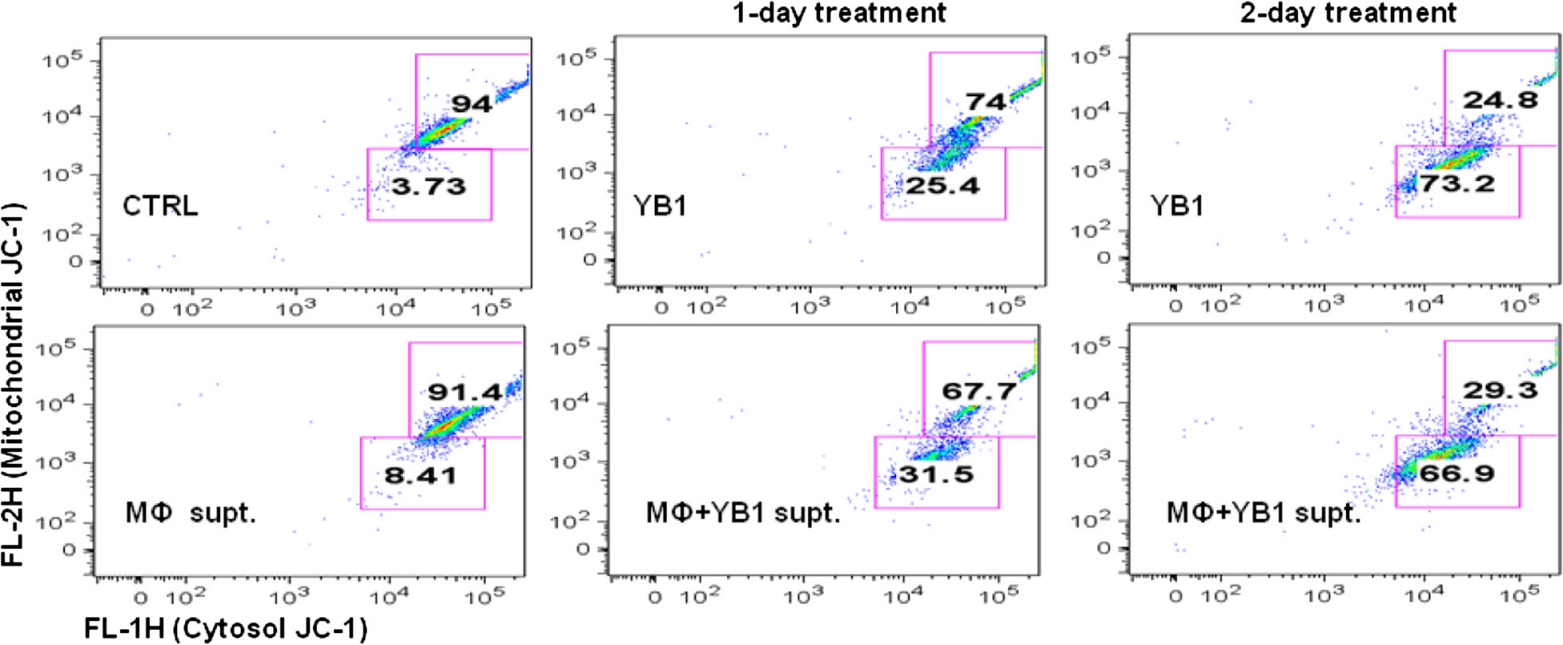
Sal-YB1 could decrease the survival of neuroblastoma via JC-1 assay. JC-1 flow cytometry indicated that the survival of neuroblastoma (gated in the *upper box*) decreased after either Sal-YB1 direct treatment or co-culture with Sal-YB1 pre-treated activated macrophages culture supernatant

**Fig. 6:**
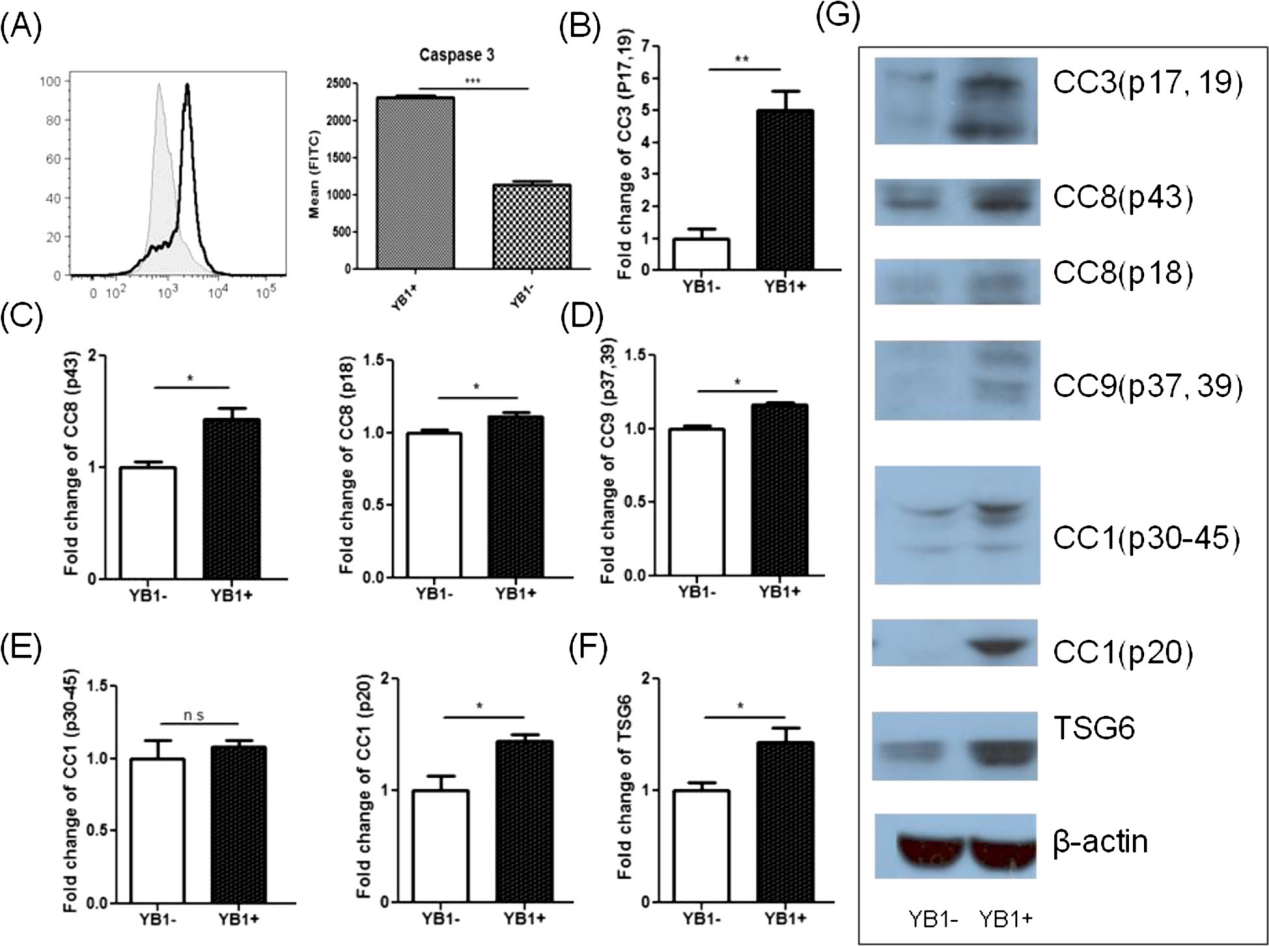
Sal-YB1 could trigger the caspase-dependent apoptosis signalling pathway. **a** YB1-treated mouse tumor tissues showed high expression of caspase 3 by flow cytometry. *Gray filled histogram* and *continuous line* represented the YB1-untreated and -treated mice, respectively. **b–f** Cleaved caspase 1, 3, 8, and 9 and TSG6 could be increased in Sal-YB1-treated tumor tissues by Western blot. **g** Western bands. The *error bars* showed means ± SEM for three nude mice in each group. *P < 0.05 and ^***^ *P* < 0.001 by an unpaired two-tailed *t* test

Using the supernatant from Sal-YB1 pre-treated macrophages or Sal-YB1 direct treatment on neuroblastoma could induce apoptosis as shown by the JC-1 assay (Fig. 5). This finding implies the involvement of the intrinsic apoptotic pathway. Sal-YB1-treated mouse tumor tissues showed high expression of caspase 3 by flow cytometry (Fig. 6a). Cleaved caspase 1 (p20) (*P < 0.05*), cleaved caspase 3 (p17, 19) (*P < 0.01*), cleaved caspase 8 (p18, 43) (*P < 0.05*), cleaved caspase 9 (p37, 39) (*P < 0.05*), and TNF-stimulated gene 6 protein (TSG6) (*P < 0.05*) could be significantly identified in Sal-YB1-treated tumor mass by Western blotting (Fig. 6b–g). Pan-caspase inhibitor could block the apoptosis of neuroblastoma, which is treated by post-activated macrophage culture supernatant (*P < 0.001*) and Sal-YB1 (*P < 0.05*) (Fig. 4e, f). These findings further supported that the TNFα by macrophages induced indirect tumor cell killing in the cancer microenvironment via caspase-dependent apoptotic pathway.

## Discussion

In the past decades, the paradigms of cancer treatments are mainly based on surgical resection, irradiation, chemotherapy, immunotherapy, and targeted therapy. There are also advocates on using alternative approaches including homeopathic therapy, hyperthermia, or some other strategies but all with doubtful impact [16, 17]. Neuroblastoma is the most common childhood extracranial solid tumor derived from neural crest cells [2, 18]. Although a minority of cases spontaneously regress, more than half of the children suffered from metastatic disease at diagnosis, and they have poor prognosis despite current therapeutic approaches [19–21].

Recently, novel treatment such as using an attenuated measles virus to patients with disseminated myeloma raised the possibility of applying biotherapy with genetically modified microorganism [8]. Such approach has been attempted, but its clinical application has been limited by low efficacy and potential adverse effects. Currently, SL7207 has been tested as a form of lived bacterial vaccine in pre-clinical studies [22–24]. Here, we proposed a novel treatment strategy by using an engineered anaerobe *Salmonella* strain Sal-YB1. Such “bio-bullet” has a survival advantage in a hypoxic environment, which is a common phenomenon within the cancer core where the cancer stem cells reside. Similar to most stem cells, cancer stem cells thrive better in a hypoxic environment. Therefore, Sal-YB1 may overcome the current limitations of conventional treatment by getting rid of the cancer cells within the hypoxic microenvironment. Sal-YB1 only replicates under a hypoxic environment and therefore is unlikely to cause serious infection in the recipient. Furthermore, any potential infection can also be easily controlled by conventional antibiotics. Compared to chemotherapy, it has the advantage of having less off-target organ toxicity. In addition, unlike measles virus therapy, the issue of immune rejection effect of *Salmonella* is unlikely as there is no routine vaccination for *Salmonella*. As in our animal model, Sal-YB1 can effectively target at the tumor mass with minimal side effect on the mice.

How the Sal-YB1 induced cell death of cancer such as neuroblastoma cells remains unknown. There may be direct or indirect effects on the cancer cells. To find out the likely influence of the host immune system on such cytotoxicity, we make use of two different types of immune-deficient mice to find out the possible involvement of the host immune system. Nude mice (deficient in T cells) and NOD-SCID mice (deficient in T, B, NK cells, and MΦ) were used in our orthotopic xenograft neuroblastoma mouse model [14, 15]. The mice were applied to mimic the induced immune-deficient state of cancer patients after chemotherapy. Despite the severe immune-deficient state of the mice, none of them died of infection. Interestingly, we noted a much higher tumor necrosis rate among nude mice than NOD-SCID mice, suggesting the contribution of non-T immune cells in the event. On the first day post-YB1 injection, we observed that all the mice turned inactive and four nude mice developed transient skin rash, suggesting transient bacteremia associated with immune activation leading to inflammation. All the mice subsequently regained their body weight 3 days after Sal-YB1 inoculation. As the treatment effect was demonstrated in the mice, the increased body weight should not be contributed by the progression in tumor burden especially compared to the control group. While we know that wild type *Salmonella* can cause infection, or even a chronic carrier state, in people with normal immunity, our findings suggested that the anaerobic Sal-YB1 is safe even if applied to these severely immune-deficient mice. But whether Sal-YB1 may be rejected by immune-competent mice remains unanswered. Testing of such an approach on transgenic mice with spontaneous neuroblastoma development may help to answer this question. However, we can adopt this approach as an adjuvant to chemotherapy. Then, the immune rejection issue will be resolved because chemotherapy can induce an iatrogenic immune-deficient state.

Referring to the bio-safety concern of potential *Salmonella*-induced environmental pollution, the excreta was screened*.* There was no *Salmonella* found in the urine of the mice. Seventy-five percent of mice showed positive Sal-YB1 in their stool, but they all turned negative within 2 weeks. The genetically modified Sal-YB1 can only proliferate under hypoxic condition or by supplementing with DAP under aerobic condition. The usual Sal-YB1 detection was by using DAP-supplemented plate in normal incubation at 37 °C. Here, we double-checked the stool by parallel DAP-free CFU test. Unlike detection rate in DAP+ plate, Sal-YB1 is nearly undetectable in DAP-negative stool culture. Therefore, the worry of *Salmonella* causing environmental pollution remains theoretical.

**Fig. 7:**
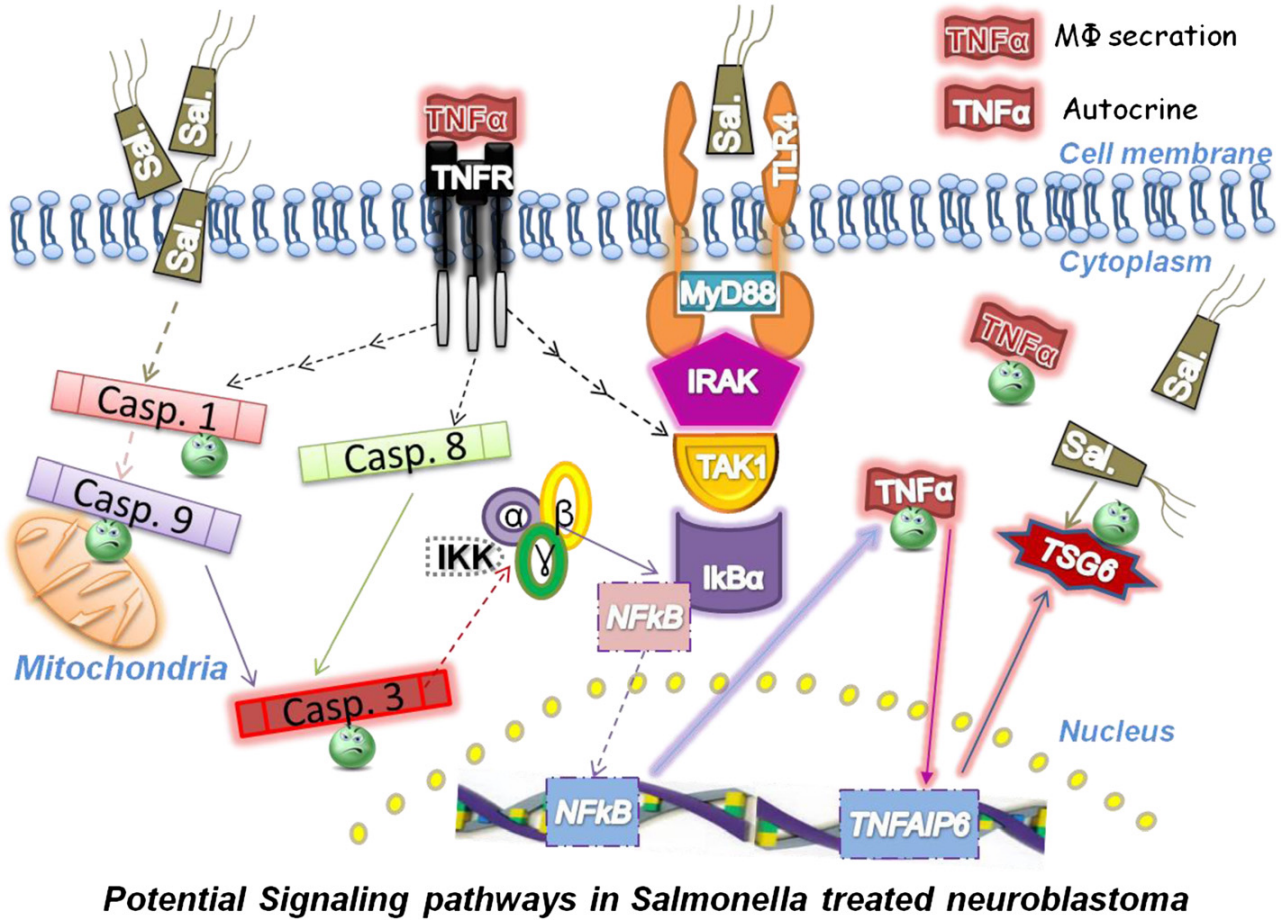
Possible mechanisms of Sal-YB1-induced cytotoxic effects on neuroblastoma. Bio-engineered *Salmonella* with the assistance of macrophages should contribute to the neuroblastoma reducing progression. TNFα release from macrophages is likely to be one of the cytotoxic components. Abbreviation: *TLR4* toll-like receptor 4, *MyD88* myeloid differentiation primary response gene 88, *IRAK* interleukin 1 receptor-associated kinase, *TAK1* TGF-β-activated kinase 1, *IκBα* nuclear factor of kappa light polypeptide gene enhancer in B-cells inhibitor alpha, *IKK* IκB kinase, *NF-kB* nuclear factor kappa B, *TNFα* tumor necrosis factor alpha, *TNFR* tumor necrosis factor receptor, *TNFAIP6* tumor necrosis factor alpha-induced protein 6, *TSG6* TNF-stimulated gene 6 protein, *Casp*. caspase

We further explored the possible mechanism of Sal-YB1-induced cytotoxicity in vivo (Fig. 7). Increased TLR4 expression may account for the resistance of mice to Gram-negative bacterial infection, for it elicits stronger immune response from the host [25, 26]. Tumor necrosis factor can enhance the lethal effect of endotoxin in mice, but there is no difference in TNFα expression in *S. typhimurium*-infected TLR4+/− mice [27, 28]. Gram-positive and -negative bacteria could both activate MyD88/IRAK/IKK/NFκB signalling pathways [29, 30]. Our data showed that Sal-YB1 could upregulate TLR4 expression in the tumor tissues. The direct cytotoxicity was observed via the activation of TLR4. The treatment effect was significantly different between nude and NOD-SCID mice. It is probably due to the number and function of B cell, NK and macrophages are relatively preserved in the nude mice [31, 32]. Our investigation suggested that the indirect anti-cancer effect of *Salmonella* was due to the activation of macrophages. To test our hypothesis in vitro, we treated the human macrophages with Sal-YB1 and found that Sal-YB1 had significant direct stimulatory effect on macrophages. Then, we collected the supernatant from the macrophages and co-cultured it with human neuroblastoma cells. The viability of neuroblastoma was significantly decreased in the group co-cultured with the supernatant derived from macrophages pre-treated with Sal-YB1. Moreover, there was a high release of TNFα from both human macrophages and mouse tumor tissues in the Sal-YB1-treated group. TNFα and pan-caspase inhibitors could both reverse the anti-neuroblastoma effect induced by macrophages. TNFα release from macrophages as suggested by our in vitro and in vivo experiments is likely to be one of the cytotoxic components found in our animal model. Our results highlighted that the anti-tumor function of Sal-YB1 therapy was mediated via TLR signalling pathway and the activation of IRAK and IκBα are determinant for TNFα release. The TNFα could further trigger the expression of caspase 1 and 8 [33, 34]. Caspase 1 could induce the mitochondria to release caspase 9 through the complex multistep process. Both caspase 8 and 9 could activate caspase 3-induced apoptosis [35–38]. On the contrary, TSG6, the negative feedback loop during the inflammatory/apoptosis network, could be upregulated by LPS and TNFα [39, 40]. These downstream apoptotic related proteins could all be detected to have enhanced expression in our nude mice tumor tissues. Thus, one of the possible mechanisms of Sal-YB1-induced cytotoxic effect on neuroblastoma may be due to the activation of macrophages by Sal-YB1.

The limitation of the current study is that the status of immunodeficient mice is not totally similar to the post-chemotherapy-treated immunosuppressed mice for they are often having neutropenia as well. Currently, there are several mouse models with spontaneous neuroblastoma development such as those with amplified *MYCN* [41] or mutated *ALK* [42]. In future study, we can make use of such models to simulate the actual clinical setting and test our approach. Our proposed possible underlying mechanisms can also be revisited and verified.

## Conclusions

In conclusion, our approach provides a new paradigm in targeting cancer cells residing within the hypoxic microenvironment. Even in mice with severe immune-deficient backgrounds such as nude or NOD-SCID, the Sal-YB1 could still induce tumor necrosis without causing severe infection. The macrophages recruited by Sal-YB1-directed therapy may play a significant role in fighting against the cancer. Even Sal-YB1 is not tumor specific, it helps to control both primary refractory tumors and metastatic tumors via direct and indirect pathways. Sal-YB1 can also serve as a therapeutic vector in carrying drugs to poorly vascularized hypoxia regions, so it may open up another new frontier in eradicating cancer in the future.

## Methods

### Culture of *Salmonella* strains SL7207 and YB1

*S. typhimurium* strain YB1 was kindly provided by Prof. JD Huang. Sal-YB1 was cultivated at 37 °C in Luria-Bertani (LB) broth (USB, Cleveland, USA) containing 25 μg/mL chloramphenicol, 50 μg/mL streptomycin (Sigma-Aldrich, St. Louis, USA), and 50 μg/mL DAP (DACO, Tokyo, Japan). OD600 was measured to determine the bacterial count. The final calculation was based on 1 OD unit of bacteria medium equivalent to 10^9^ bacteria.

### Maintenance of human neuroblastoma cell lines and macrophages

SK-N-LP/luciferase cell line passages 7–10 were cultured in Dulbecco’s modified Eagle’s medium-high glucose (DMEM-HG), supplemented with 100 U/mL penicillin, 100 mg/mL streptomycin (Invitrogen, New York, USA), 10 % heat-inactivated fetal bovine serum (Hyclone, Logan, USA), and 1000 μg/mL G418 (Roche, Mannheim, Germany). Macrophages were isolated from healthy human peripheral blood mononuclear cell and kept in RPMI 1640 (Invitrogen, New York, USA) medium containing 5 % autologous serum and 1 % penicillin-streptomycin. The medium was renewed every other day. Normal incubator was supplied with a humidified atmosphere at 37 °C with 5 % CO_2_, but the hypoxic chamber was precalibrated with less than 0.5 % O_2_.

### Establishment of orthotopic neuroblastoma mouse model and treated with Sal-YB1

Four-week-old male nude and NOD-SCID mice were purchased from the Laboratory Animal Unit of the University of Hong Kong with the approval of the Hong Kong Department of Health and Committee on the Use of Live Animals in Teaching and Research of the University of Hong Kong (CULATR 2587-11 and 2887-12). Mice were routinely anesthetized and disinfected for abdominal operation. With the surgical operation microscope, 2E+05 SK-N-LP/luciferase cells diluted in 50 % Matrigel (BD Biosciences, San Diego, USA) were administered directly to the fat pad of the mice’s left side adrenal glands. By intraperitoneal injection of the luciferin (Invitrogen, New York, USA), the xenograft condition (no more than 4000 mm^3^) was monitored via Xenogen in vivo imaging system bi-weekly*.* The ROI was generated automatically, and its value was normalized under the luminescence interval 12,256 to 2.4512E+07. Three weeks post-tumor cells transplantation, the mice were divided into two groups according to the similar tumor ROI value. Sal-YB1 (5E+07 CFU) dissolved in 100 μL PBS was inoculated via the tail vein of the mice, whereas the control group was treated with 100 μL PBS only. The body weight and temperature were recorded and also the urine and stool were collected daily for 2 weeks. Then, the mice were sacrificed by an overdose of pentobarbital. Tissues of the tumor, heart, lung, spleen, liver, kidney, brain, and spinal cord were harvested for *extro-vivo* experiments. The length, width, and height of the tumor were measured for the calculation of the final xenograft volumes, using formula: 4/3 × π (Length × Width × Height)/8.

### Histology

Around 1–2 cm^3^ tissues of the tumor, heart, lung, spleen, liver, kidney, brain, and spinal cord were resected and immediately immersed in 4 % paraformaldehyde for overnight fixation. Paraffin-embedded block was sectioned into approximately 4 μm slices and mounted on polylysine pre-coated slide. H&E staining was performed to examine the overall structure and necrosis of the tissues. *Salmonella* distribution was detected with rabbit anti-*Salmonella* (Abcam 35156, Cambridge, UK). Bound primary antibody (1:700) was detected with horseradish peroxidase-conjugated goat anti-rabbit secondary antibody (1:8000) and then developed in DAB solution (DACO, Tokyo, Japan). Pictures were taken under a ×200 light microscope.

### CFU test

LB agar plates containing 25 μg/mL chloramphenicol and 50 μg/mL streptomycin were prepared with ±50 μg/mL DAP supplement. Ten microliters of urine, blood, and bile were pipettied on DAP+ plate. Stool and tissues of the tumor, heart, lung, spleen, liver, kidney, brain, and spinal cord were weighed, homogenized, serially diluted in PBS, and plated on the previous descried LB agar plates. *Salmonella* colonies were counted after 12–18 h incubation at 37 °C.

### *Salmonella* targeting neuroblastoma/macrophages in vitro

SK-N-LP/Luciferase cells were cultured in antibiotic-free DMEM medium the day before they were co-cultured with *Salmonella* at a ratio of 1:500. *Salmonella* was eliminated by adding 500 μg/mL gentamycin-supplemented medium for 20–30 min 2–2.5 h later. Then, the cells were replenished with DMEM containing 100 μg/mL gentamycin under a normoxia incubator or a precalibrated hypoxic chamber (O_2_ < 0.5 %). Similarly, the DMEM was changed to RPMI1640 for macrophages, and the supernatant of macrophages was also collected after 2 days. Fifty percent supernatant of the YB1 pre-treated macrophages was supplemented to the SK-N-LP/luciferase cells for another 2 days.

### XTT cell proliferation assay

SK-N-LP/luciferase cells were seeded in a 96-well plate (around 4000 cells/well). After overnight culture, SK-N-LP/luciferase cells were pre-treated with DMSO, TNFα antibody (Sigma-Aldrich, St. Louis, USA), caspase 1 (Biovision, San Diego, USA), and pan-caspase (Calbiochem, Darmstad, Germany) inhibitors for 2 h, respectively. The viability of neuroblastoma with *Salmonella* or macrophages supernatant treatment was observed by XTT kit (Roche, Mannheim, Germany) for three continuous days or after 2 days, respectively. The optical density was measured using a microplate reader at wavelength 450 nm.

### JC-1 assay

For the JC-1 (BD, San Diego, USA) analysis, the experiment involved one no-treatment negative control arm and two experimental treatment arms. The two treatment arms involved 1 or 2 days treatment, respectively, as illustrated in the following. The no-treatment control served as a control for comparison of both 1- and 2-day treatment with Sal-YB1. The data were analyzed by using FlowJo 8.8.2.

**Table:** 

Day 1	Seed cells	Well 1	Well 2	Well 3
Day 2	Treatment to well 3	–	–	+
Day 3	Treatment to well 2	–	+	+
Day 4	Flow (JC-1)	Control group	1-day treatment group	2-day treatment group

### Flow cytometry

Mouse tissues of the tumor, heart, lung, spleen, liver, kidney, brain, and spinal cord were weighed, homogenized, and filtered with 70 μm cell strainer on ice. Suspension containing around 5E+04 single cells was prepared to perform flow cytometry within 1 h. The apoptosis of neuroblastoma triggered by Sal-YB1 was analyzed by annexin V/PI (BD, San Diego, USA). Annexin V+/PI−, annexin V+/PI+, and annexin V−/PI+ cells are divided as early apoptosis group, late apoptosis group, and dead group, respectively. The TLR4 expression induced by Sal-YB1 was detected by anti-human CD284 (TLR4) PE (HTA 125), using mouse IgG2a K isotype control PE (eBM2a) (Ebioscience, San Diego, USA). The count of TLR4 positive expression rate was gated according to the isotype control. The FITC active caspase 3 apoptosis kits were purchased from BD Pharmingen. The data were analyzed by using FlowJo 8.8.2.

### ELISA assay

Approximately 1.5 g tumor tissues were homogenized, filtered, and centrifuged at 4 °C. Concentration of TNFα in the collected supernatant was measured using mouse TNFα ELISA kit (Ebioscience, San Diego, USA) according to the manufacturer’s instructions. Macrophages were seeded in a 24-well plate (around 5E+05 cells/well). The supernatant of macrophages with/without Sal-YB1 treatment was detected by human TNFα ELISA kit (R&D Systems, Minneapolis, USA). The optical density was measured using a microplate reader at wavelength 450 nm with correction at 570 nm.

### Cell lysis, SDS-PAGE, and Western blotting

Approximately 5 mg tumor tissues were weighed, homogenized, and lysed directly with radio immune precipitation assay buffer for 2 h with constant agitation in cold room. Lysates were clarified by centrifugation for 20 min at 12,000 rpm at 4 °C, and the protein concentrations were quantificated by protein assay kit (Bio-Rad, Hercules, USA). SDS-PAGE and Western blotting were performed by standard techniques. Spectra multicolor prestained protein ladder (Thermo, New York, USA) was used as the size standard in gel electrophoresis and Western blotting. Primary rabbit polyclonal antibodies (1:1000) of IRAK (Millipore 06-872, Bedford, USA), IκBα (Santa Cruzs c-203, Santa Rosa, USA), and caspase 1, 3, 8, and 9 (CST, Danvers, USA), and mouse monocolonal antibody of TSG6 (R & D Systems 259820, Minneapolis, USA) in PBS-Tween 20 (Bio-Rad, Hercules, USA) containing 5 % bovine serum albumin (Sigma-Aldrich, St. Louis, USA) were prepared. Diluted β-actin (1:8000) (CST 4967, Danvers, USA) was compared as internal control. Subsequently, 1:4000 secondary anti-rabbit antibody was used, and immunocomplex was visualized by enhanced chemiluminescence (Pierce, Chicago, USA). The density of the protein bands was calculated by Quantity One (Bio-Rad, Hercules, USA).

### Statistical analysis

Statistical analysis was performed with GraphPad Prism 5 (GraphPad Software, San Diego, USA). Differences between groups were analyzed by the unpaired, two-tailed Student *t* test. All data was presented as the means ± SEM. *P* value less than 0.05 was considered as statistically significant.

## Abbreviations

Caspase: cysteinyl aspartate specific proteinase
CC: cleaved caspase
DAP: diaminopimelic acid
ELISA: enzyme-linked immunosorbent assay
H&E: hematoxylin and eosin
I: inhibitor
IHC: immunohistochemistry
IκBα: nuclear factor of kappa light polypeptide gene enhancer in B-cells inhibitor alpha
IKK: IκB kinase
IRAK: interleukin 1 receptor-associated kinase
IVIS 100: in vivo imaging system Xenogen 100
MyD88: myeloid differentiation primary response gene 88
MΦ: macrophage
NFkB: nuclear factor kappa B
NK: natural killer cell
Sal-YB1: *Salmonella* YB1
TAK1: TGF-β-activated kinase 1
TLR4: Toll-like receptor 4
TNFAIP6: tumor necrosis factor alpha-induced protein 6
TNFα: tumor necrosis factor alpha
TNFR: tumor necrosis factor receptor
TSG6: TNF-stimulated gene 6 protein
